# A lightweight cow mounting behavior recognition system based on improved YOLOv5s

**DOI:** 10.1038/s41598-023-40757-7

**Published:** 2023-10-13

**Authors:** Rong Wang, Ronghua Gao, Qifeng Li, Chunjiang Zhao, Weihong Ma, Ligen Yu, Luyu Ding

**Affiliations:** 1 Information Technology Research Center, Beijing Academy of Agriculture and Forestry Sciences, Beijing, 100097 China; 2 College of Information Engineering, Northwest A&F University, Yangling, 712100 China; 3 National Engineering Research Center for Information Technology in Agriculture, Beijing, 100097 China

**Keywords:** Biomedical engineering, Electrical and electronic engineering, Animal behaviour

## Abstract

To improve the detection speed of cow mounting behavior and the lightness of the model in dense scenes, this study proposes a lightweight rapid detection system for cow mounting behavior. Using the concept of EfficientNetV2, a lightweight backbone network is designed using an attention mechanism, inverted residual structure, and depth-wise separable convolution. Next, a feature enhancement module is designed using residual structure, efficient attention mechanism, and Ghost convolution. Finally, YOLOv5s, the lightweight backbone network, and the feature enhancement module are combined to construct a lightweight rapid recognition model for cow mounting behavior. Multiple cameras were installed in a barn with 200 cows to obtain 3343 images that formed the cow mounting behavior dataset. Based on the experimental results, the inference speed of the model put forward in this study is as high as 333.3 fps, the inference time per image is 4.1 ms, and the model mAP value is 87.7%. The mAP value of the proposed model is shown to be 2.1% higher than that of YOLOv5s, the inference speed is 0.47 times greater than that of YOLOv5s, and the model weight is 2.34 times less than that of YOLOv5s. According to the obtained results, the model proposed in the current work shows high accuracy and inference speed and acquires the automatic detection of cow mounting behavior in dense scenes, which would be beneficial for the all-weather real-time monitoring of multi-channel cameras in large cattle farms.

## Introduction

The health status of animals is expressed through various external representations, such as their posture and behavior. In dairy cows, the mounting behavior directly reflects whether the dairy cow is in estrus. In addition, the mounting behavior of dairy cows is monitored using sensors or computer vision systems, which facilitates the improvement of management strategies and economic efficiency^1,2^. However, the methods based on sensor technology require the installation of multiple sensors on animals, which might affect the growth of the animal and cause stress responses in them^3,4^. Surveillance video could replace traditional human observation. Techniques including image processing and video analysis may be utilized to detect animal behavior and analyze the interaction between individual animals to understand better the living habits of animals^5^. These image-processing technologies are applied widely in the field of smart farming.

Traditional image segmentation algorithms classify images based on decision rules, which are applied to the decision of cow estrus^6^. The mounting behavior is defined as a cow following another cow. The region of interest obtained from each frame could be used to determine if a cow is mounting by calculating the change in the length of the moving cow’s body^7^. In addition, the geometric and optical flow features in the video could be analyzed to determine whether the cow is mounting^8^. Chung et al.^9^ introduced an approach to detecting cow estrus using a surveillance video captured from an overhead field of view. The afore-stated method first detects the motion region in the video. Then it analyzes the information related to direction, amplitude, and history of the motion behavior to identify whether mounting has occurred, which enables the automatic detection of cows in estrus. Such methods have confirmed the feasibility of implementing non-contact cow mounting recognition based on image processing. However, in all such methods, the selection of the features of mounting is manual, which renders the extraction of the mounting features incomplete, thereby leading to low accuracy.

The advancements in deep learning have allowed the application of convolutional neural networks (CNNs) to adequately extract image features, which has been used extensively in agriculture. For instance, using pattern identification, the YOLO network was applied to determine the cow number^10^. Another study used the combination of optical flow and CNNs to detect lameness in cows^11^. Porto et al. developed an approach based on the Violee-Jones algorithm to detect the feeding and standing behavior of cows with a multi-camera video recording system to acquire a panoramic top-view image of the barn area^12^. Although these deep learning-based methods achieve high accuracy, they pay less attention to the lightweight level of the model. Zhang et al. proposed a sow behavior detection algorithm using the lightweight network MobileNet to detect three typical sow behaviors—drinking, urination, and mounting^13^. Although the model uses MobileNet as the backbone network to make it lighter, its feature extraction capability is poor, which can result in a decrease in model accuracy. Achour et al. identified the feeding behavior and identity of 17 Holstein cows using a camera to capture images of the cows’ backs^14^. Li et al. successfully developed three deep cascaded convolutional neural network models, respectively, the convolutional pose machine model, the stacked hourglass model, as well as the convolutional heatmap regression model, for a robust estimation of cattle-pose with the RGB images captured based on real cattle farm situations^15^. Wu et al. put forward a method on the basis of YOLOv3 and relative step feature vectors to distinguish between lame and non-lame cows. At first, the authors employed the YOLOv3 algorithm to determine the legs of the cows in each image frame, and later the relative step-length feature vectors of the cow’s front and rear legs were constructed on the basis of the leg coordinates. In the end, using long-term and short-term memory networks, the lame and non-lame cows were identified^16^. Jiang et al. improved the accuracy of cow lameness recognition by enhancing the time range of the single-stream long-time stream convolutional network model^11^. Ayadi et al. trained CNNs by adopting surveillance cameras to capture the images of all cow poses to effectively identify regurgitation and other behaviors in these cows^17^. These studies have validated the feasibility of using deep learning to recognize dairy cow behavior, but the YOLOv3 series algorithms have a large number of parameters and slow inference speed. When monitoring the behavior of a group of dairy cows, multiple cameras are required to cover the entire movement area of the cows, and there are high requirements for the lightweight nature and inference speed of the model. The current algorithms are unable to meet the needs of monitoring the behavior of a group of dairy cows. In another study, a combination of CNN and LSTM allowed the recognition of the daily behavior of cows in complex environments^18^. Another study reported the development of efficient Net-LSTM models for recognizing the lying, standing, walking, drinking, and feeding behaviors of a single cow^19^, which could be adopted for health state perception and disease prevention in cows. These deep learning methods have exhibited high accuracy in livestock behavior recognition and served as a reference and feasible basis for cow mounting behavior recognition. However, to achieve high accuracy, all of these models exhibit improved feature extraction capability through increased model depth, which leads to numerous model parameters and a high consumption of hardware resources.

In order to perform the detection of cow mounting with geometric and optical flow, Guo et al. put forward a method. The region's geometric and optical flow features were extracted and adopted to train a support vector machine to classify the detected region as mounting or non-mounting^8^. However, optical flow requires calculating the motion information of each individual pixel, which is very time-consuming and not suitable for large-scale dairy farming. Wang et al. applied the YOLOv3 model to identify cow estrus behavior with a detection speed of 31 frames per second (fps)^20^. However, the cow mounting datasets used in the above studies were captured at ideal angles and contained only one or a few cows, causing the recognition scenarios to be relatively simple, the number of model parameters to be large, and the recognition speed and accuracy to remain poor, with a scope of much improvement. Wang et al. used YOLOv5 to detect cow mounting behavior in a dairy herd with an inference speed of 71 fps^21^. However, since a free-range farming scenario is characterized by an extensive range, numerous cows, and several cameras, the inference speed and the lightness of the model had to be maintained high. Owing to the huge data and the limitation of hardware resources during practical applications, a further lightweight backbone network is needed to enhance the inference speed of the model and reducing the consumption of hardware resources. However, reducing the number of parameters of the model to render it further lightweight would also decrease the accuracy of the model. Therefore, the current research is becoming focused on how to design a novel network model that would allow the cow crawling behavior recognition model to achieve high accuracy and lightweight aspect simultaneously.

In order to address the above concerns, the present study aims to propose a lightweight model for the rapid recognition of cow mounting behavior based on improved YOLOv5. First, the attention mechanisms, depth-separable convolution, and inverted residual structure are used for designing a lightweight backbone network to lower the number of model parameters and enhance the inference speed of the model. Different feature extraction modules are designed to broaden the depth and width of the backbone network, aiming to ensure a strong feature extraction capability. Next, the lightweight feature extraction module is designed by integrating an attention mechanism and ghost convolution, aiming to optimize the prediction branch of the model. Finally, a rapid recognition model of cow mounting behavior using the lightweight backbone network and the lightweight feature extraction module is constructed to improve the speed and accuracy of cow estrus behavior recognition in free scenes. This paper is organised in the following manner: (1) Data collection and analysis which describe data sources, data analysis, and preprocessing. (2) Methods that describe the architecture of YOLOv5s, reconfiguration of the backbone network, and enhancement module based on the efficient attention mechanism and Ghost convolution (3) Experimental results and discussion that describe the experimental details and result, and (4) Conclusion that discussion and future scope of the work.

## Data collection and analysis

### Data sources

**Figure 1:**
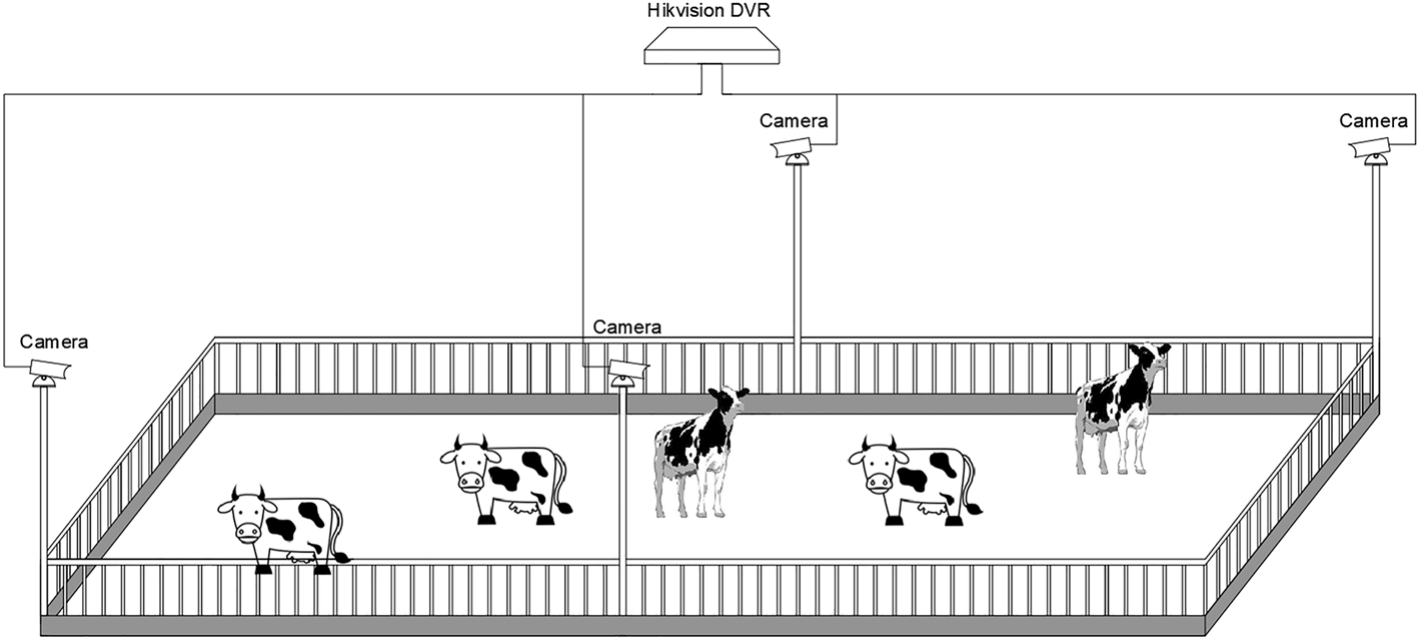
Schematic diagram of the installation of the estrus data acquisition equipment.

The cow experimental data were gathered in Beijing Dadi Qunsheng Dairy Cows Breeding Base, in Yanqing District, Beijing, China. This young cowshed was 70 m × 26 m in size, with cameras installed at the height of 4.5 m to have the field of view cover the entire young cowshed. As a high-definition infrared night vision camera (DS-2CD3T46WDV3-I3, Hikvision, China), the used camera had a focal length of 6 mm, a pixel number of 4 million, a resolution of 2560 × 1440 (pixels), as well as a frame rate of 25 fps. The images were captured during the day and at night, with all images being RGB color images. As shown in Fig. 1, the captured video was kept in a hard-disk video recorder (NVR, HIKVISIONDS-8832N-K8, Hikvision, Hangzhou, China). The videos were recorded every day, for the entire period of 24 h, between 26 September 2021 and 6 October 2021, totaling 10 days of video data. The extraction of cow mounting images was performed from the cow surveillance video. The video is encoded in H256 format and stored in a mechanical hard drive.

### Data analysis and preprocessing

**Figure 2:**
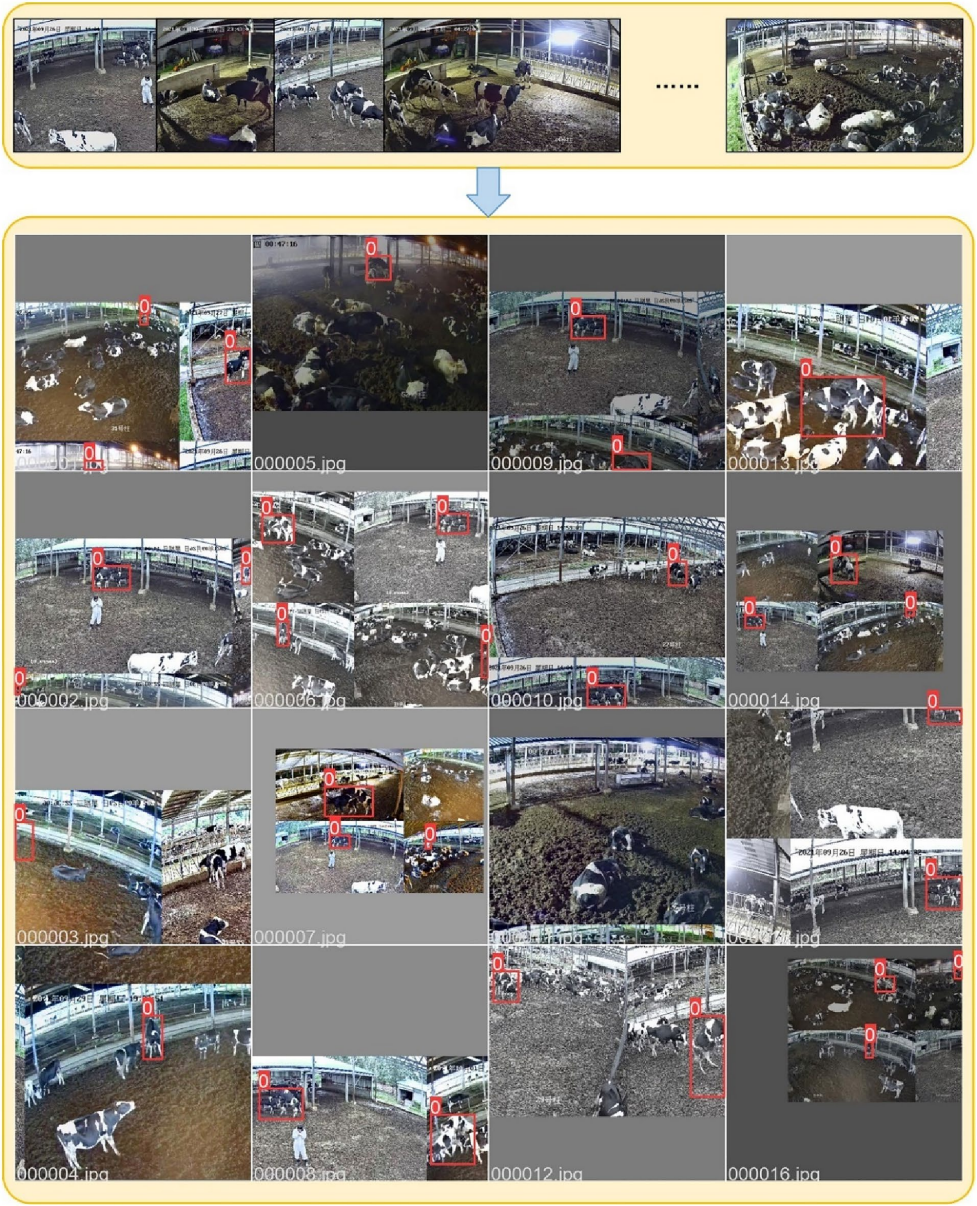
The results of data enhancement.

The LabelImg software was adopted for manually marking the mounting regions in the images to create a cow mounting behavior dataset. To ensure that the mounting videos of the training set and the test set came from various scenes, the dataset was divided in a ratio of 8:2, with the extraction of the 2668 images from the first 115 mounting videos applied to be the training set, whereas the 675 mounting images extracted from the last 29 mounting videos were applied to be the test set. This also allowed for further objectively evaluating the generalization of the model used in dense scenes (Fig. 2).

The cow mounting behavior dataset was collected on a free-range cattle farm, and Fixed-position cameras were employed to capture cow behavior from different distances (Table 1). The dairy cow mounting images had multiple scales, angles, and complex lighting interference. Therefore, the mosaic enhancement method was adopted to stitch, randomly rotate, translate, scale, and crop the original image. Finally, the brightness, chromaticity, and contrast of the mosaic-enhanced images were modified randomly, and 50% of the images were randomly chosen and flipped vertically to acquire the enhanced cow mounting training set.

**Table 1:** Cow mounting datasets.

Growth periods	Number of videos	Number of images	Image enhancement methods	Image resolution
Training set	115 videos	2668 images	Mosaic enhancement	2560 × 1440
Test set	29 videos	675 images	–	2560 × 1440
Total	144 videos	3343 images	–	2560 × 1440

## Methods

### YOLOv5

**Figure 3:**
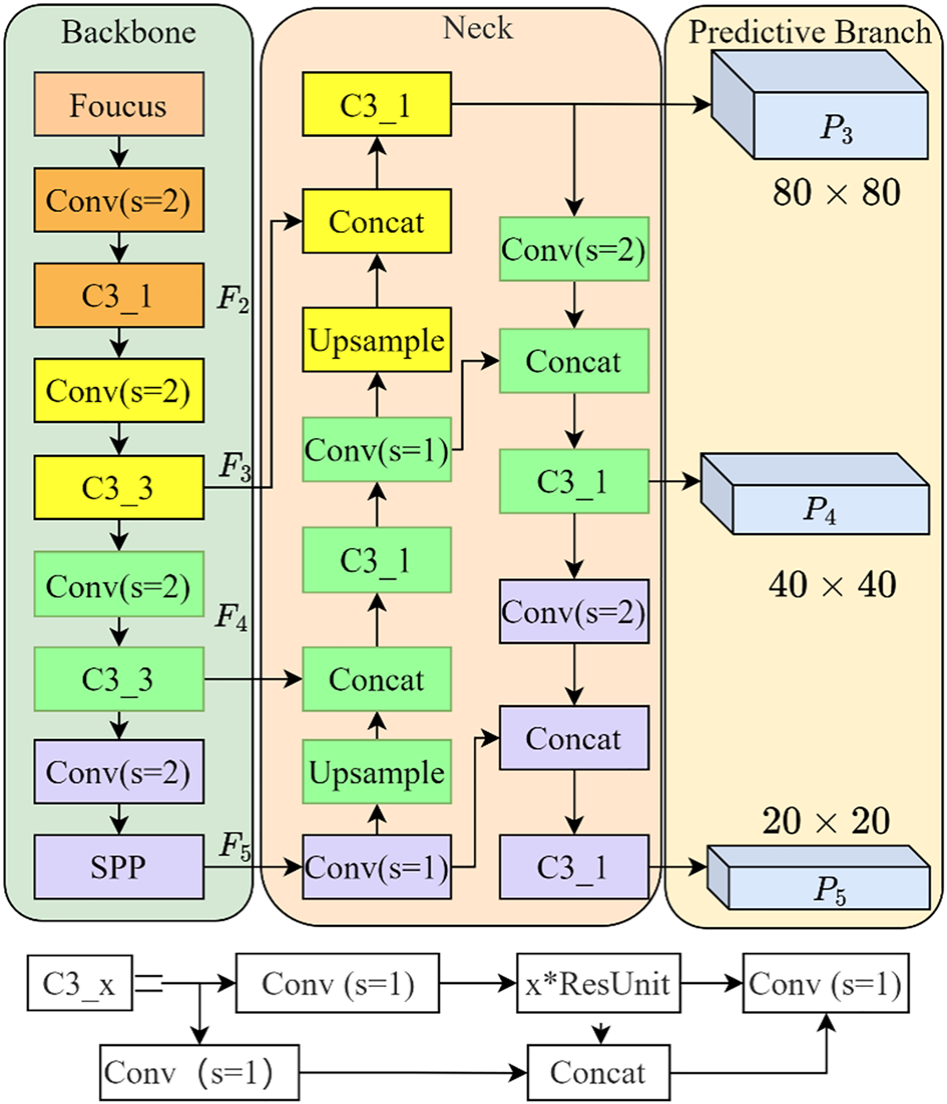
The structure of the YOLOv5s model.

Ultralytics proposed the YOLOv5 family of algorithms, which included YOLOv5s, YOLOv5m, YOLOv5l, and YOLOv5x. The network structure increased in depth, width, and the required computational resources, sequentially in this series of algorithms (Ultralytics, 2020). The overall network architecture of YOLOv5s, as depicted in Fig. 3, could be broadly divided into three parts—the backbone network, the neck network, and the prediction branches.

For YOLOv5s, its backbone network consists primarily of Conv(s = 2), C3_x, Focus, as well as spatial pyramid pooling (SPP)^22^. Through interval sampling, the Focus module enables the RGB image (640 × 640 × 3 input size) slicing to yield 4 feature images, each of which is 320 × 320 × 3 in size. The subsequent step is splicing the 4 foregoing images into feature maps with a size of 320 × 320 × 12 in the channel dimension. Finally, a feature map with a size of 320 × 320 × 16 is generated as the output. This focus module efficiently lowers the loss of image information during downsampling, decreases the amount of calculation, and enhances the calculation speed. Conv(s = 2) is a convolutional layer with a step size of 2. The layer implements feature map downsampling. The C3_x module contains x residual structures, which are the main building blocks of the backbone network. Introducing the SPP module into YOLOv5s facilitates the information fusion of local and global features.

The neck network is inspired by feature pyramids (FPN) and path aggregation networks (PAN). It is constructed as the FPN + PAN structure, in which FPN employs a top-down approach to fuse the features extracted by the backbone network at different layers, while PAN adopts a bottom-up approach to fuse the semantic information of the different layers outputted by FPN. Consequently, the neck network allows for the fusion of both shallow and deep-feature information, aiming to improve the performance of the detector.

Three feature maps with the sizes of 20 × 20 pixels, 40 × 40 pixels, and 80 × 80 pixels, respectively, are generated in the prediction branch, which allows for the prediction of cows at different scales. The standard non-maximum suppression (NMS) operation is adopted for filtering out the redundant prediction frames to obtain the prediction findings of the model.

YOLOv5s currently has many issues, and its backbone network mainly consists of standard convolution and residual structures, which significantly limits the model's lightweight capability. Therefore, lighter convolutions should be used to construct a lightweight backbone network. When the backbone network is relatively lightweight, it can lead to a decrease in the model's feature extraction capability. To balance the trade-off between accuracy and lightweight, the feature extraction capability of the Neck and Predictive branch should be enhanced to achieve a higher accuracy for the lightweight network.

### Reconfiguration of the backbone network

#### Feature extraction module

**Figure 4:**
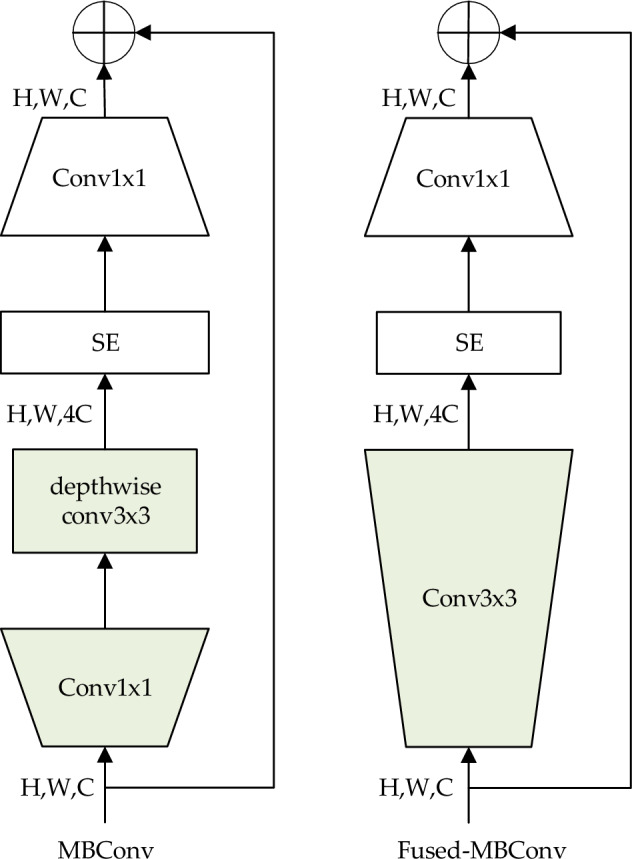
The structure of the MBConv module and the Fused-MBConv module.

The present study, inspired by EfficientNetv2, redesigned the lightweight backbone network to replace the backbone network in YOLOv5s^23^. Two types of inverted residual modules are designed in the new lightweight backbone network: the Fused-MBConv module and the MBConv module. To extract the shallow features of the image, the Fused-MBConv module can be employed, while the MBConv module is applied with the aim of extracting the deep features of the image. Figure 4 illustrates the structure.

The Batch Normalization (BN) layer and the Squeeze-and-Excitation (SE) module are introduced in both MBConv and Fused-MBConv modules. While the BN layer performs a normalization operation on the input values of each hidden layer neuron in the deep network, the SE module enhances the network’s capability of extracting features from the cows’ mounting images. First, the MBConv module up-samples the input feature maps using the 1 × 1 convolutional, BN layer, and SiLU activation functions. Next, the 3 × 3 depth-separable convolutions are used for extracting image features, which can significantly lower the number of operations and parameters. Subsequently, the important features in the image can be extracted using the SE channel attention module, and the feature weights are recalibrated. Finally, the feature map is downscaled to H × W × C using the 1 × 1 convolutional layer. The Fuse-MBConv module uses the 3 × 3 standard convolution rather than the 1 × 1 convolution and the 3 × 3 depth-separable convolution in MBConv. Therefore, Fused-MBConv is a convolutional layer merged with the MBConv module, which compresses the model scale and improves the computing speed as well as the feature extraction ability of the shallow network.

**Figure 5:**
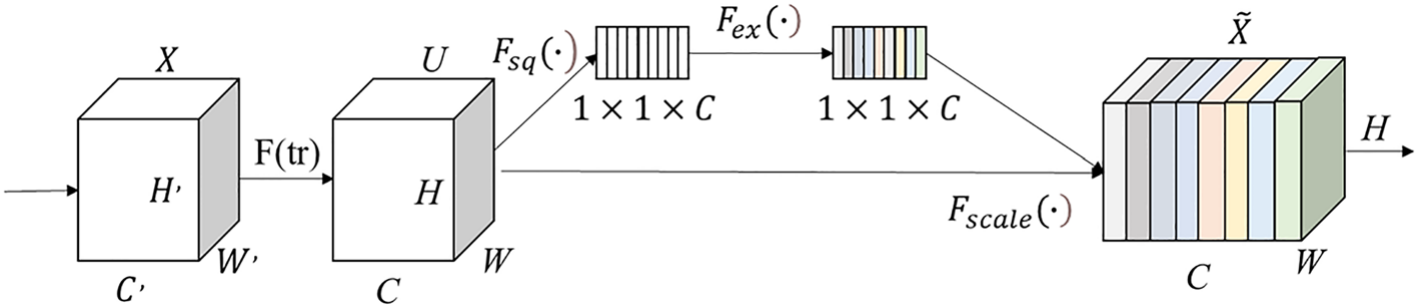
The schematic diagram of the SE module.

Figure 5 displays the schematic structure of the SE module. The feature map *U*,$U \in R^{H \times W \times C}$ is obtained after the feature map X is convolutionally transformed *F(tr)*, following which the features are recalibrated using the SE module. The SE module initially compresses the feature map *U* into a $1 \times 1 \times C$ format using the compression operation $F_{sq} \left( \cdot \right)$. This operation generates the channel descriptors by aggregating the feature maps (*H* × *W*) across spatial dimensions. The function generates an embedded globally-distributed channel feature response that integrates the global receptive domain information for use by the other layers. $F_{ex} \left( \cdot \right)$ is the stimulus operation that functions to filter the aggregated global features. Next, the set of modulation weights for each channel is generated using a self-selecting gate function (sigmoid) and used to the feature map *U* to output a new feature map. The slope of the ReLU activation function is not smooth. Moreover, the slope has to be defined before training and updated in training, which is convenient only in tasks with sparse gradients. This drawback causes the model to be less capable of learning tasks with dense gradients. Cows are dense in natural scenes, and the replacement of the ReLU function with the SiLU function, which is both smooth and non-monotonic, allowed for further complete extraction of the detailed characteristics in the image. Let the output of the upper layer be x. Then, the SiLU activation function can be denoted to be Eq. (1) provided below.

1 $$SiLU\left(x\right)=x\cdot Sigmoid\left(x\right)$$

2 $$Sigmoid\left(x\right)=\frac{1}{1+\mathrm{exp}\left(-x\right)}$$

#### Lightweight backbone network structure

The present study proposes a lightweight backbone network with the structure details presented in Table 2. This network is a novel, smaller, and rapid model with greater advantages in terms of training speed and parameter efficiency. The replacement of the original feature extraction network in YOLOv5s with the proposed lightweight backbone network would lower the number of parameters of the YOLOv5s model and enhance the inference speed.

**Table 2:** The structural parameters of the lightweight backbone network.

Layers	Network layer	Step	Channels	Blocks	Params
1	CBS	2	16	1	464
2	Fused-MBConv	1	16	1	2336
3	Fused-MBConv	2	24	1	10,928
4	Fused-MBConv	1	24	1	23,280
5	Fused-MBConv	2	32	1	24,064
6	Fused-MBConv	1	32	1	41,280
7	MBConv	2	16	1	7840
8	MBConv	1	16	2	5824
9	MBConv	2	24	1	5136
10	MBConv	1	24	3	26,496
11	MBConv	2	32	1	6688
12	MBConv	1	32	5	49,600
13	SPPF	–	512	1	34,336

### Feature enhancement module based on the efficient attention mechanism and ghost convolution

While the reconstructed lightweight backbone network lowers the number of parameters of the model and also enhances the effectiveness of the parameters, it causes feature loss to a certain degree. Therefore, in the present study, a new feature enhancement module is designed using an efficient channel attention mechanism, ghost convolution, and residual network and then added to the Neck network to enhance the model’s capability, aiming to extract the deep semantic features of cow mounting images.

#### Analysis of the ghost convolution parametric number and computational effort

**Figure 6:**
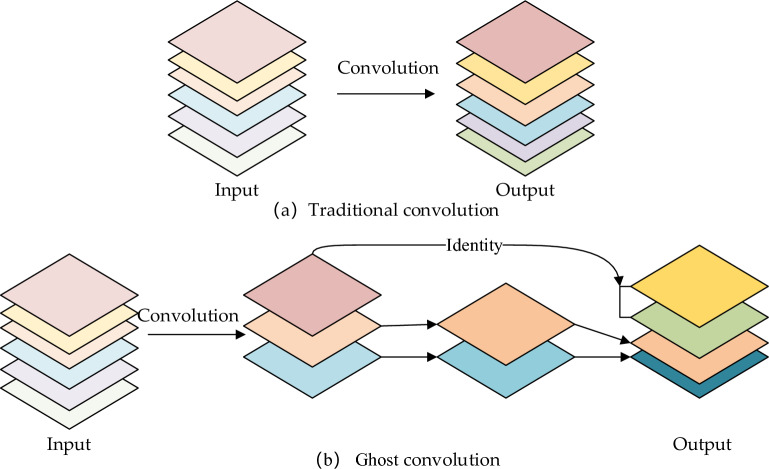
Comparison of traditional convolution and Ghost convolution.

Figure 6 displays the operation processes of the traditional convolution and Ghost convolution. The traditional convolution is classified into the following two steps on the basis of ghost convolution: Step 1 employs a small amount of traditional convolution to generate *m* original feature maps; step 2 adopts the* m* original feature maps for generating *s* ghost feature maps after a linear operation, and finally, ghost convolution outputs *m* × *s* feature maps after the completion of the first two steps.

When the output is *n* feature maps, the number of parameters is ${p}_{1}$ and ${p}_{2}$ for the traditional convolution and Ghost convolution networks, respectively.

3 $${p}_{1}=n\cdot c\cdot k\cdot k$$

4 $${p}_{2}=\frac{n}{s}\cdot c\cdot k\cdot k+\left(s-1\right)\cdot \frac{n}{s}\cdot d\cdot d$$

In the above expressions, *c* represents the number of channels in the input image, and $k\cdot k$ suggests the convolution kernel size. Besides, the difference between the two parameter quantities is expressed in Eq. (5), provided below.

5 $$\frac{{p}_{1}}{{p}_{2}}=\frac{n\cdot c\cdot k\cdot k }{\frac{n}{s}\cdot c\cdot k\cdot k+\left(s-1\right)\cdot \frac{n}{s}\cdot d\cdot d }\approx \frac{s\cdot c}{s+c-1}\approx s$$

The computation of the model using the traditional convolution and the ghost convolution is ${q}_{1}$ and ${q}_{2}$, respectively, and is calculated using Eq. (6) provided below.

6 $${q}_{1}=n\cdot {h}^{\prime}\cdot {w}^{\prime}\cdot k\cdot k$$

7 $${q}_{2}=\frac{n}{s}\cdot {h}^{\prime}\cdot {w}^{\prime}\cdot c\cdot k\cdot k+\left(s-1\right)\cdot \frac{n}{s}\cdot {h}^{\prime}\cdot {w}^{\prime}\cdot d\cdot d$$

The ratio of ${q}_{1}$ and ${q}_{2}$ is obtained using Eq. (8).

8 $$\frac{{q}_{1}}{{q}_{2}}=\frac{n\cdot {h}^{\prime}\cdot {w}^{\prime}\cdot k\cdot k }{\frac{n}{s}\cdot {h}^{\prime}\cdot {w}^{\prime}\cdot c\cdot k\cdot k+\left(s-1\right)\cdot \frac{n}{s}\cdot {h}^{\prime}\cdot {w}^{\prime}\cdot d\cdot d }\approx \frac{s\cdot c}{s+c-1}\approx s$$

where, ${h}^{\prime}$ and ${w}^{\prime}$ suggest the height and the width of the feature map generated by Ghost convolution, respectively; $d\cdot d$ refers to the size of the convolution kernel for linear operation; and s <  < c. According to Eqs. (5) and (8), with *k* and *d* being equal, the number of parameters obtained using Ghost convolution is nearly 1/*s* of the number of parameters obtained using conventional convolution.

**Figure 7:**
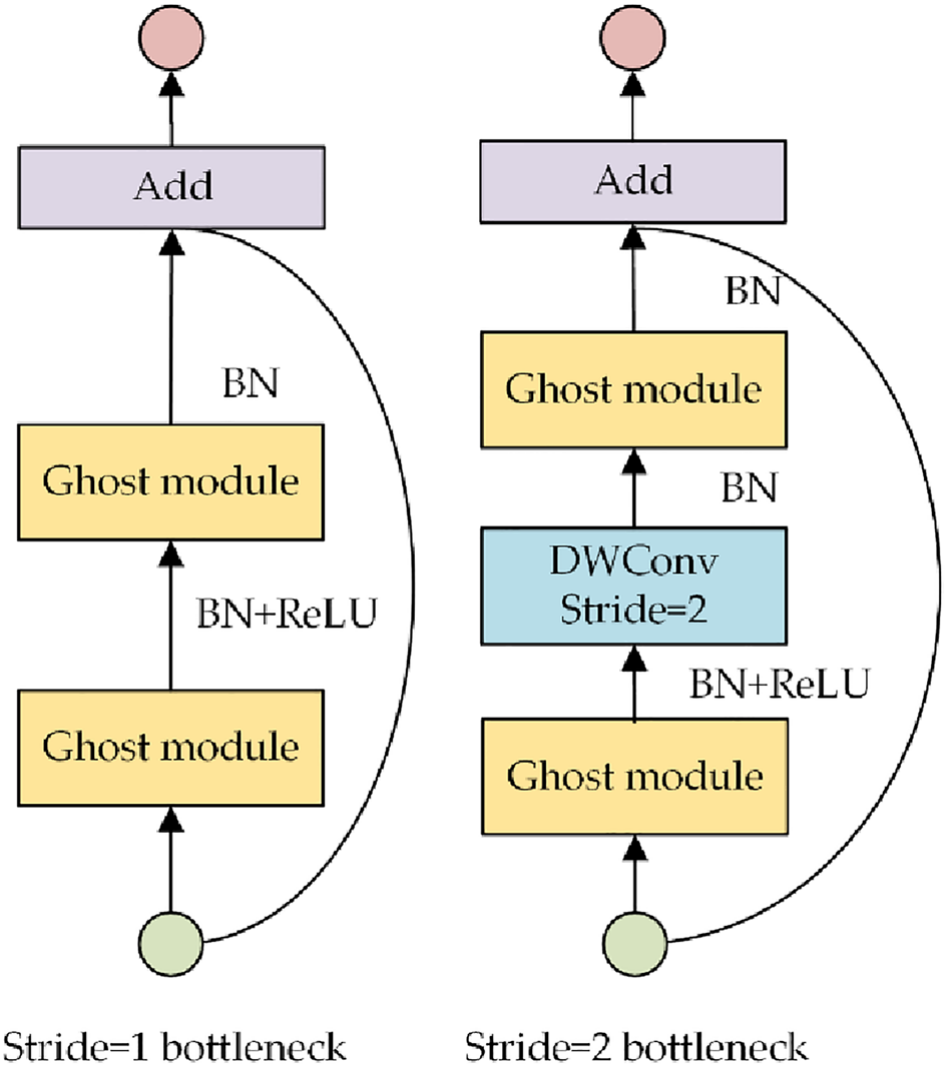
The structure of Ghost Bottleneck.

Based on Fig. 7, the structure of the Ghost Bottleneck using Ghost convolution is illustrated. When the step size is 1, the Ghost Bottleneck contains two Ghost convolution modules. At step size 2, a depth-separable convolution is supplemented between the two Ghost convolution modules, aiming to increase the module’s feature extraction capability.

#### Lightweight cow mounting behavior recognition model

**Figure 8:**
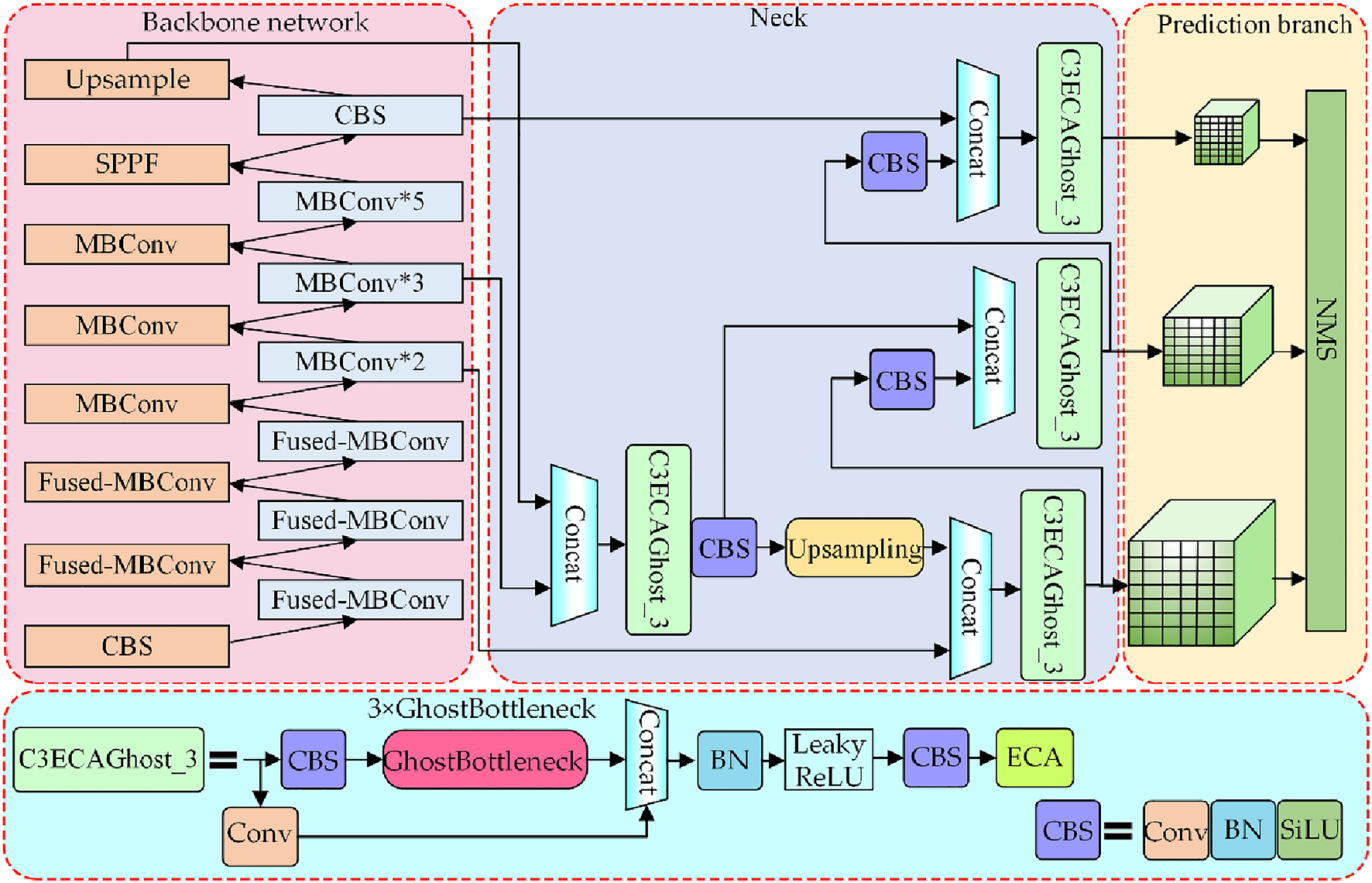
The architecture of the proposed lightweight cow mounting behavior recognition model.

The feature enhancement module constructed through the fusion of Ghost bottleneck, efficient channel attention (ECA), and residual network extract deep semantic information and is named C3ECAGhost_3. Together, these modules form a lightweight cow mounting behavior recognition model, the structure of which can be found in Fig. 8.

Figure 8 illustrates the architecture of the lightweight cow mounting behavior recognition model. On the foundation of YOLOv5s, the model uses the reconstructed lightweight backbone network, described in section "Reconfiguration of the backbone network", to extract image features. In contrast, C3ECAGhost_3 is used for improving the neck network of YOLOv5s to obtain a lightweight cow mounting behavior recognition model. The C3ECAGhost_3 structure is similar to that of C3_x in YOLOv5s. The feature maps are input separately into the two branches for convolution, and the three ghost bottlenecks are added to the first branch to extract the detailed features of the image. Next, the two branch feature maps are concated, and the concated feature maps are input into the convolution layer and the ECA module.

**Figure 9:**
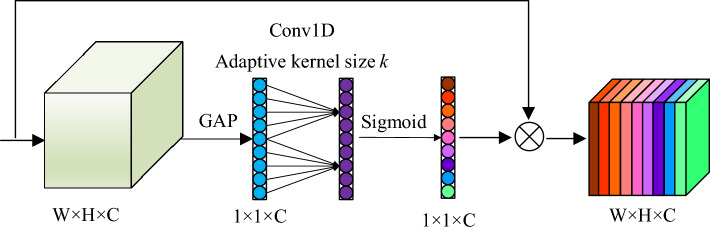
The structure diagram of the ECA module.

Figure 9 depicts the specific structure of the ECA module. The feature map is output after the convolution transformation as ${u}_{c}(i,j)\in {R}^{W\times H\times C}$, in which W, H, and C denote the width, height, and channel dimensions. The attention module uses a compression operation to compress the feature map *U* into the 1 × 1 × *C* format. The operation can aggregate the features across spatial dimensions (*H* × *W*), generate channel descriptors, and obtain aggregate features $F({u}_{c})\in {R}^{C}$ without dimensionality reduction. The EAC attention module utilizes global average pooling (GAP) to acquire aggregated features and later uses 1D convolution to enable all its channels to share the same learning weights. The efficient attention mechanism does not downscale the original channel features, and the high-dimensional channels form longer-range interactions that are not restricted to the local receptive domain of the convolutional response. This enables enhancing and recalibrating the important features in the upper and lower layers.

To confirm the performance of the proposed model, the model was assessed based on qualitative and quantitative perspectives. During the qualitative evaluation, the performance of the model was assessed by discovering the differences between the detection findings of YOLOv5 and the other methods using human eyes, that is, through contrasting the accuracy of locating the object box, detecting the existence of missed or false detections. Based on the quantitative evaluation, precision (P), recall ®, average precision (AP), and mean average precision (MAP) were applied to be the evaluation indicators. In addition, the scope of P and recall was [0,1]:

9 $$R={TP}_{1}/({TP}_{1}+{FN}_{1})$$

10 $$P= {TP}_{1}/({TP}_{1}+{FP}_{1})$$

${TP}_{1}$, ${FP}_{1}$, and ${FN}_{1}$ indicate the number of true positives, false positives, and false negatives, respectively. Besides, a curve known as the P–R curve is drawn in accordance with the recall and P of each class, and the area under the curve represents the AP, representing the AP of each class. We can find the calculation formula in Eq. (11):

11 $$AP= {\int }_{0}^{1}P(r)dr$$

*M* represents the number of classes, and *MAP* denotes the average of all classified APs. It indicates the higher the value of MAP, the better the detection capability of cow mounting behavior. Besides, the calculation can be made with Eq. (12):

12 $$mAP=\frac{{\sum }_{c=1}^{M}AP(c)}{M}$$

## Experimental results and discussion

### Experimental parameter settings

We used a 16-GB NVIDIA Tesla P100 GPU for training. Meanwhile, a DL algorithm training platform was built using the Ubuntu 16.0 operating system, Python 3.8, and PyTorch 1.7.1. Additionally, the CUDA API version 10.1 and the CuDNN version 8.0.5 were employed. The initial learning rate in the training process was determined to be 0.01. The cosine annealing strategy was adopted to reduce the learning rate. The input image size, batch size, and the number of epochs were defined to be 640 × 640 (pixels), 40, and 200, separately. During the model training process, data augmentation techniques such as random cropping, random flipping, and color transformations from Section "Data analysis and preprocessing" are used to enhance the dataset, improving the model's generalization ability. The cosine annealing learning rate scheduling strategy is then employed, causing the learning rate to increase initially and then decrease during training, aiding model convergence. Additionally, label smoothing techniques are used during training, reducing the confidence of labels, making the model pay more attention to samples with lower confidence, thus improving the model's generalization ability.

### Model training results

**Figure 10:**
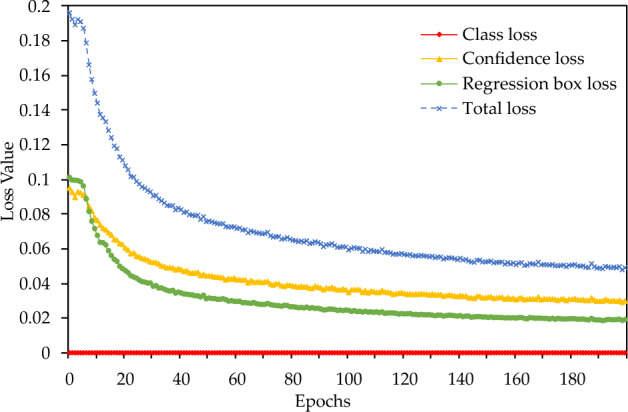
The trend of each loss function in the training process.

**Figure 11:**
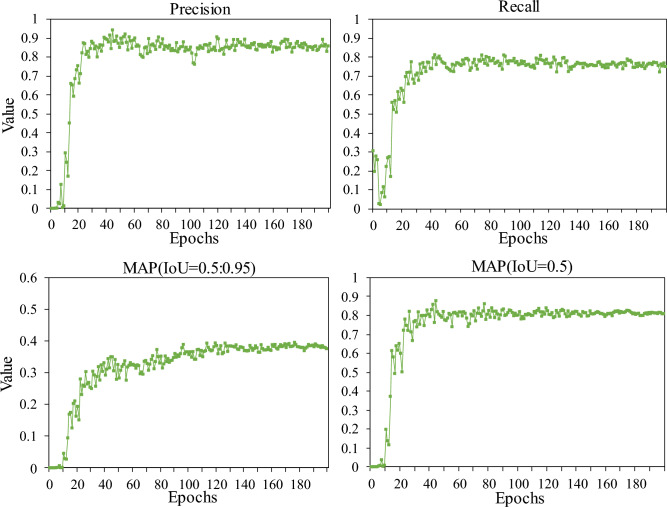
The test results obtained using the model for the test dataset during training.

At first, the improved lightweight cow mounting behavior recognition model was trained with the dataset provided in Table 1. After each round of training, the recognition effect of the model was tested. The change process of each loss function is illustrated in Fig. 10. The test results for the test dataset in the training process are presented in Fig. 11.

The model contains four types of loss functions. The convergence of the model could be evaluated based on the trend of the loss functions in the training process. Figure 10 illustrates the trend of each loss function within the training process. As the number of training rounds increases, all loss functions gradually decrease and later tend to oscillate within a certain interval. Whether a loss function tends to stabilize or not indicates whether the model would converge or not, respectively. In the present study, the loss function of the model tends to become stable gradually, which confirms that the model has learned the best results with the increasing number of training epochs. Besides, when the number of training epochs is increased further, the loss function no longer exhibits a decrease, and the effect of model learning no longer changes. Therefore, the model achieves good convergence after 200 rounds of training, with the confidence error and regression error converging to approximately 0.006, the target category loss function converging to 0, and the overall error converging to approximately 0.012. The low value of the loss function of the model confirms that the model has a strong learning ability for the characteristics of cow mounting.

Following each epoch of training, the model was evaluated on the test set for the recognition effect of cow mounting behaviors, and the test results were quantified using different evaluation metrics. Figure 11 illustrates the change process of each evaluation index of the model within the test set in the training process. The main evaluation indices of the model are classified into four categories: precision, recall, MAP (IoU = 0.5:0.95), and MAP (IoU = 0.5). Precision is concerned with the proportion of cow mounting behavior in the detection results expressed as a percentage of images. Recall is concerned with the number of cow mounting behaviors detected in the test set. MAP measures the average accuracy of each category when the IoU has different thresholds; the higher the value of MAP (IoU = 0.5:0.95), the better the generalization ability of the model. Additionally, in the present study, as the number of training epochs increases, each evaluation index of the model gradually increases and then stabilizes. Precision and recall finally stabilize within the intervals of 0.8–0.9 and 0.9–1.0, respectively, which confirms that the model detects the cow mounting behavior with high accuracy. At IoU = 0.5, the MAP value of the model stabilizes in the interval of 0.8–0.9, and the model with the highest MAP is saved as the optimal model. In an unconstrained environment, the MAP of the optimal model is 87.9%, which achieves the expected results.

**Figure 12:**
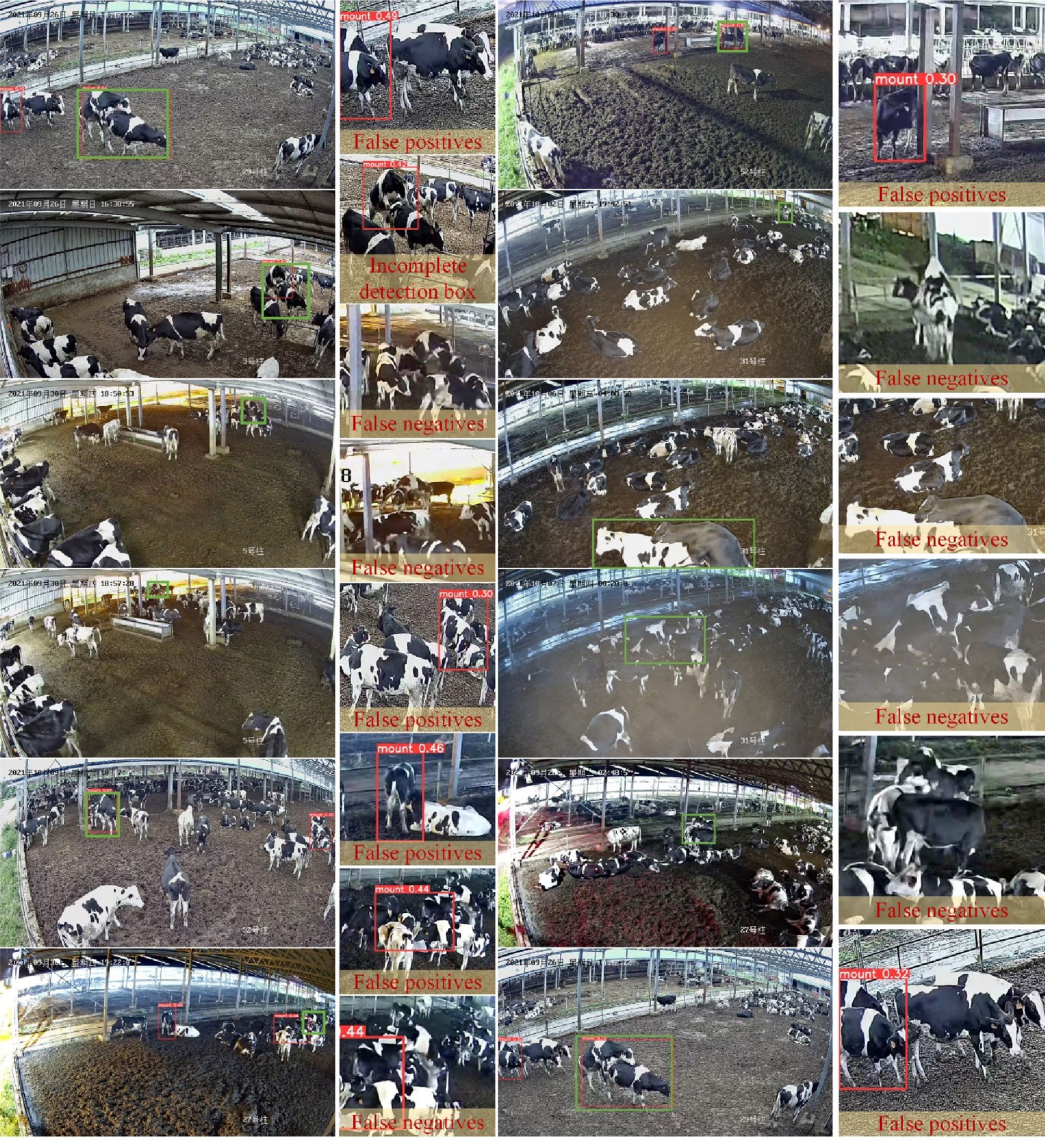
Examples of incorrectly detected cow mounting behavior.

Select image samples where the model has made recognition errors for analysis, as shown in Fig. 12. The model labeling other behaviors of cows as mounting behavior is referred to as false positives, while labeling mounting behavior of cows as other behaviors is referred to as false negatives. The examples of false positives and false negatives in the model's recognition are shown in Fig. 12. The red boxes represent the model's detection results, while the green boxes represent the manually annotated labels. When two cows are standing crosswise, both human eyes and the model are prone to mistaking it for a mounting behavior. False positives can also occur when cows are in the middle of a group or obstructed by buildings. Translation: Additionally, factors such as blurry images, foggy weather, obstructions, small scale, or incomplete capture can all contribute to false negatives in the model's recognition, leading to missed detections of cows mounting behavior. In the context of cow movement, despite the presence of small scale and occlusion, the majority of cows mounting behavior can still be detected by the model. Moreover, the model exhibits low confidence in incorrectly identifying such behaviors, which demonstrates the strong recognition capabilities of the model described in this paper. Additionally, based on the confidence levels of false positives and false negatives, different thresholds can be adjusted according to specific scenarios to improve the accuracy of cow mounting behavior recognition.

### Comparison of results among different models

The current mainstream object detection models are Faster RCNN, YOLOv3, YOLOv4, YOLOv5 and YOLOv8. The mainstream object detection models were trained from scratch using the training set (Table 1) and then tested for the effect of cow mounting behavior on the test set. As displayed in Table 3, the obtained test findings with different models. The mAP (IoU = 0.5), inference time per image, inference speed, and weight size were used for evaluating the test results of each model. The model proposed outperforms Faster RCNN, YOLOv3, YOLOv4, YOLOv5s, YOLOv5m, and YOLOv8s in terms of the inference time per image, inference speed, and weight size. In addition, the value of mAP (IoU = 0.5) obtained for the proposed model is lower than that obtained for YOLOv4 and much higher than that obtained for Faster RCNN, YOLOv3, YOLOv5s, YOLOv5m, and YOLOv8s. The mAP of the proposed model is 87.7%, and the inference time of each image is 3 ms, implying that it could infer 333.3 images per second. Therefore, the detection accuracy and the detection speed of the proposed model fulfill the requirements of practical applications. Meanwhile, to reduce the consumption of hardware resources by the model, a series of lightweight improvements were introduced to the proposed model, leading to a final model weight size of just 4.1 MB, which significantly lowers the number of model parameters and realizes a lightweight cow behavior detection model.

**Table 3:** The cow mounting behavior recognition results acquired with various models.

Model	mAP (IoU = 0.5)/%	Time/ms	Speed/fps	Weight/MB	Parameters/M
Faster RCNN	83.6	91.7	10.9	315.0	–
YOLOv3	83.9	14.3	69.9	117.7	61.5
YOLOv4	93.2	44.1	22.7	244.4	64.4
YOLOv5s	85.6	4.4	227.3	13.7	7.1
YOLOv5 m	86.3	7.9	126.6	40.4	21.1
YOLOv8s	85.5	4.0	250.0	21.46	11.1
Ours	87.7	3.0	333.3	4.1	2.0

The current object detection models contain Faster R-CNN^24^, YOLOv3, YOLOv4^25^, YOLOv5 series^26^ and YOLOv8^27^. YOLOv5 shows the best performance and speed reported so far. Therefore, in the present study, the YOLOv5 series model was employed to assist in enhancing the inference speed and the lightweight aspect of cow estrus detection.

**Figure 13:**
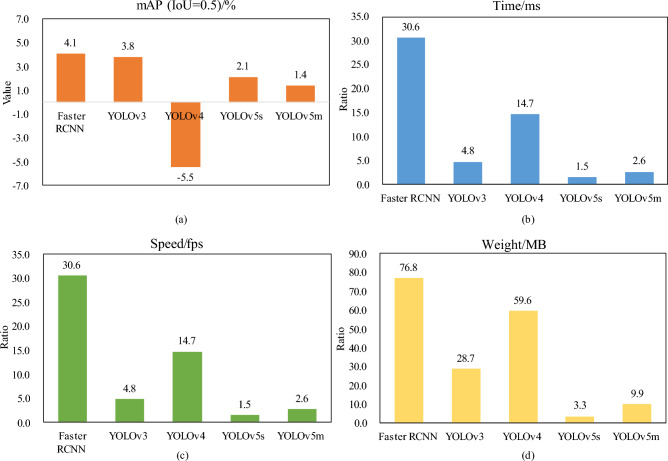
The comparison of the model in the present study with the other existing models.

To further analyze the impact of the proposed improved model, the detection results of the proposed model and those of the other models used for comparison were analyzed from different perspectives. Figure 13 presents the comparison of the proposed model with the other existing models. Figure 13a presents the results of subtracting the values of mAP from the proposed model and the values of mAP from the other models. Figure 13b presents the ratio of the inference time of other models for each image and the inference time of the proposed model for each image. Figure 13c presents the ratio of the inference speed of the proposed model to the inference speed of the other models.

The value of mAP (IoU = 0.5) of the proposed model is lower than that of the mAP (IoU = 0.5) of YOLOv4, although the inference speed of the former is 14.7 times greater than that of YOLOv4. In addition, the weight size of YOLOv4 is 59.6 times that of the weight size of the proposed model. The inference times of the Faster RCNN, YOLOv3, YOLOv5s, YOLOv5m and YOLOv8s models are 30.6, 4.8, 1.5, 7.9 and 1.3 times greater, respectively, than the inference time of the proposed model. Therefore, it is clear that the proposed model achieves a lighter weight by lowering the number of parameters. Figure 12d presents the ratio of the weight size of the other models to the weight size of the proposed model. The model weight sizes of Faster RCNN, YOLOv3, YOLOv4, YOLOv5s, YOLOv5 m and YOLOv8s are 76.8 times, 28.7 times, 59.5 times, 3.3 times, 9.9 times and 5.2 times, respectively, the weight size of the proposed models. In summary, the current work improved the YOLOv5s to finally obtain a lightweight cow mounting behavior recognition model, which improves the mAP value by 2.1% and also greatly lowers the size of the model weights and the inference speed, thereby achieving the desired results. Therefore, compared to the existing target detection models, the model put proposed in the current work better fulfills the demand for the real-time recognition of cow mounting behavior in a free-range farming environment.

**Figure 14:**
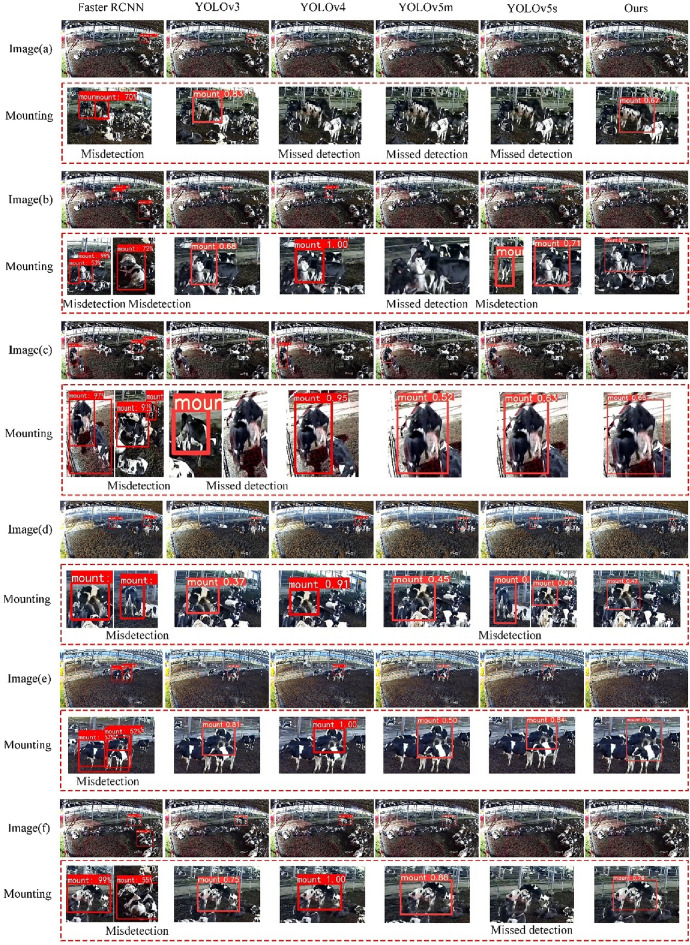
Visual analysis of the detection results obtained with the use of different models.

A visual analysis of the test findings acquired using different models is illustrated in Fig. 14. In addition, the detection findings of six models for six images are depicted in the figure. The six images contain the different scales of cow mounting behavior and a larger number of cows. The multi-scale and complex herd backgrounds raise a greater challenge for cow mounting behavior recognition. Faster RCNN exhibits the highest misdetection rate and frequently identifies aggregated cows as mounting behavior, which is a vital cause of its low accuracy. YOLOv4, on the other hand, has the highest accuracy and fewer misdetections. However, a greater number of missed detections occur for dense scenes, such as Image (a), in which small-scale cow mounting behaviors are far from the camera. In addition, the detection findings of the YOLOv3 and YOLOv5 models also involve the phenomenon of missed recognition of mounting behavior and misevaluation of cow gathering as cow mounting. In contrast, the accuracy of the model proposed in the current work is higher in detecting small and medium objects in the images (a–f), and the recognition effect is better relative to that of the other models. The model visualization effect is consistent with the conclusions obtained from Table 3 and Fig. 13, which further verifies the effectiveness of the proposed model.

### Comparison of the different improvement approaches

In the present study, based on YOLOv5s, a novel lightweight backbone network was designed to replace the backbone network of YOLOv5s and introduce feature enhancement modules on the basis of the efficient attention mechanism and Ghost convolution, aiming to strengthen the feature extraction ability and the inference speed of the model. To investigate each improvement module introduced on the model recognition results, ablation experiments are designed using the variable control method to obtain different improved models. The improved models are then trained using the same training method. Table 4 presents the comparison findings of the different improved models.

**Table 4:** Comparison results of the different improved models.

Model	Model	mAP (IoU = 0.5)/%	Time/ms	Speed/fps	Weight/MB
Model 1	YOLOv5s	85.6	4.4	227.3	13.7
Model 2	YOLOv5s + lightweight backbone network	82.8	2.9	344.8	6.02
Model 3	YOLOv5s + lightweight backbone network + C3ECA	85.8	3.0	333.3	6.02
Model 4	YOLOv5s + lightweight backbone network + C3ECAGhost	87.7	3.0	333.3	4.1

Four improved models were obtained using different optimization methods. The effects of these improved models were tested on the test set after training using the training set. Table 4 presents the comparison findings of the different improved models on the test set. Among the mainstream target detection models, YOLOv5s is a lightweight model with fewer model parameters. After training on the cow mounting dataset, the weight size of the YOLOv5s model is 13.7 MB, with an accuracy of 85.6% and 227.3 images detected per second. However, in practical applications, the limited hardware resources have to process a larger amount of surveillance data in real time, which requires a higher inference speed and a greater lightweight aspect of the model. Therefore, in the current work, a novel lightweight backbone network is designed to replace the backbone network in YOLOv5s, and Model 2 is acquired. After training, the mAP of Model 2 using the test set is 82.8%, and the weight is 6.02 MB. The inference speed of Model 2 is 117.5 images per second faster than that of Model 1, which indicates great improvement in the inference speed of the model. In the lightweight backbone network, the depth-separable convolution in the MBConv module converges gradually in the shallow network. In contrast, the Fused-MBConv module without depth-separable convolution is used in shallow networks for shallow semantic feature extraction to enhance the convergence speed of the model. The combination of MBConv and Fused-MBConv, in addition to extracting further detailed features from the image, improves the convergence speed of the model. The weight of Model 1 is twice that of Model 2, although the mAP of Model 2 is 2.8% lower than that of Model 1, which does not exactly match the expectation of a cow farm. All models recognize cow mounting behavior mainly through cow image feature extraction. The number of cows contained in the images collected in the present study is large, and the identification of the mounting behavior of the cows hidden in the herd is difficult. Therefore, the model requires to have a high feature extraction ability. Therefore, the present study introduces an efficient attention mechanism to design the CSECA module to optimize the neck network for feature enhancement in Model 3. The mAP value of Model 3 is 85.8%, which is 0.2% and 3% higher than that of Model 1 and Model 2, respectively. The weight of Model 3 is nearly the same as that of Model 2 and only half of the weight of Model 1, which, besides achieving a lightweight model, also maintains the accuracy of the model. To further enhance the accuracy in detecting cow climbing behavior and the lightweight aspect of the model, the traditional convolution in C3ECA is replaced with ghost convolution in the present study to obtain the C3ECAGhost module for enhanced feature extraction. Model 4 is then obtained by introducing the C3ECAGhost module into Model 3. After training, the mAP value of Model 4 is 87.7%, and the weight size is 4.1 MB. Model 4 infers 333.3 images per second. The mAP value of Model 4 is, accordingly, improved by 2.1% compared to that of YOLOv5s, the weight is just half of the weight of YOLOv5s, and the inference speed is increased by 106 images per second. In summary, the lightweight cow mounting behavior detection model designed in the present study has high accuracy and inference speed and consumes fewer hardware resources. Compared to the existing object detection models, the model proposed in the current work is more suitable for detecting cow mounting behavior in large cattle farms in real time.

### Comparison with other studies

The results of the comparison of the present study with other livestock behavior detection studies are presented in Table 5. Recent studies on cow behavior detection have been conducted only with small numbers of cow populations. In contrast, no study has been reported on cow mounting behavior detection for large cow populations. In separate studies, Fuentes et al.^29^ and Lodkaew et al.^31^ used the slower YOLOv3 and YOLOv4 networks combined with the I3D and ResNet50 networks, respectively, to achieve cow mounting behavior detection. The results presented in Table 3 demonstrate that the detection speed and the lightness of the model proposed in the present study are better than those of the YOLOv3 and YOLOv4 models, and the cow crawling behavior detection accuracy of the proposed model (87.7%) is shown to be higher than the corresponding accuracy of the models reported in the literature^24,25^. The weight of the proposed model is just 4.1 MB, which is lighter than the models reported in other studies, as presented in Table 5. The accuracy of identifying estrus behavior in cows in dense herds is also higher for the proposed model, which enables better detection results.

**Table 5:** Comparison of the different livestock behavior detection models.

Studies	Year	Species	Research contents	Objects	Method	Accuracy (%)
Li et al.^28^	2021	Goat	Multi-behavior recognition	A dairy goat	AlexNet + ResNet50 + Vgg16	85.6
Fuentes et al.^29^	2020	Cow	Multi-behavior recognition	27 cows	YOLOv3 + I3D	85.6
Li et al.^30^	2022	Cow	Multi-behavior recognition	A cow	MiCT	91.8
Guo et al.^8^	2019	Cow	Mounting detection	10 cows	Computer vision	90.9
Fuentes et al.^29^	2020	Cow	Mounting detection	27 cows	YOLOv3 + I3D	82.1
Lodkaew et al.^31^	2023	Cow	Mounting detection	3 cows	YOLOv4 + ResNet-50	83
Our	2022	Cow	Mounting detection	200 cows	YOLOv5s	87.7

## Conclusion

Most of the existing studies on cow mounting behavior recognition have used complex models to identify the mounting behavior of cows in sparse cow populations, which does not allow for maintaining high accuracy when the number of cows is large. In addition, complex models consume greater amounts of hardware resources. Therefore, the present study put forwards a lightweight cow mounting behavior detection model based on YOLOv5s for 200 cows, which achieves reduced consumption of hardware resources while maintaining a high level of accuracy of detection. A lightweight backbone network is designed in the present study to decrease the number of parameters of the model and enhance the model’s ability to extract shallow features. Next, a feature enhancement module (C3ECAGhost_3) is designed to fuse the college attention mechanism, residual structure, and Ghost convolution to extract deep semantic information. According to the model performance evaluation results, the proposed lightweight cow mounting behavior recognition model has a mAP value of 87.7%, a model weight size of 4.1 MB, and an inference speed of 333.3 images per second. Therefore, the performance of the proposed model is much superior to that of the mainstream object detection models. Moreover, the model proposed in the current work outperforms the other models reported in previous studies in terms of the accuracy and inference speed of cow mounting behavior recognition in dense herd cows. Therefore, it can be established that the proposed lightweight model exhibits high accuracy and high inference speed in recognizing cows’ mounting behavior while also reducing the consumption of hardware resources.

The large range of movement of cows poses a significant challenge for computer vision technology used in smart farming. In order to assist in intelligent cow farming, it is necessary for cameras to cover the entire barn. This leads to the model having to simultaneously process data from multiple camera feeds, which presents a significant challenge in terms of speed and hardware resource consumption. In future research, there will be a greater focus on developing lightweight models with very high accuracy. Both the architecture and modules of the models need to be as lightweight as possible while maintaining a high level of accuracy. This is necessary in order to apply the algorithms effectively in large-scale farming operations.

## Data Availability

Permissions were obtained from the cattle farm owners for carrying out the study in the farms. This study was a non-contact recognition of cow estrus behaviour and therefore the animals in this study were not touched. The datasets used and/or analysed during the current study available from the corresponding author on reasonable request (Ronghua Gao). The animal use protocol listed below has been reviewed and approved by the Ethics Committee, Northwest A&F University, China.
